# Robert Edwards: the path to IVF^☆^

**DOI:** 10.1016/j.rbmo.2011.04.010

**Published:** 2011-08

**Authors:** Martin H Johnson

**Affiliations:** The Anatomy School, Department of Physiology, Development and Neuroscience and The Centre for Trophoblast Research, Downing Street, Cambridge CB2 1HW, UK

**Keywords:** Edwards, genetics, history, infertility, IVF

## Abstract

The early influences on Robert Edwards’ approach to the scientific research that led to human IVF are described. His interest as a graduate student in the genetics of early mammalian development stimulated him later to investigate whether the origins of human genetic diseases such as Down, Klinefelter and Turner syndromes might be explained by events during egg maturation. This clinical problem provided the most powerful stimulus to achieve both oocyte maturation and fertilization *in vitro* in humans. Indeed, preimplantation genetic diagnosis was his main goal until he met Patrick Steptoe in 1968. A re-evaluation of his meeting with Steptoe suggests that initially Steptoe’s laparoscopic skill was of interest for its potential to solve the sperm capacitation problem. Steptoe’s impact on Edwards was twofold. First, Steptoe’s long-held interest in infertility raised this application of IVF higher in Edwards’ priorities. Second, Steptoe offered a long-term partnership, in which oocyte collection without in-vitro maturation was a possibility. The professional criticism generated by their work together encouraged Edwards to pursue a deliberate programme of public education about the issues raised and to challenge and develop professional bioethical thought and discourse about reproduction.

The early life and career of Robert Edwards are described and re-evaluated in the light of documentary evidence. His early interest in the genetics of development provided the major motivation behind his goal of achieving IVF in humans. Through this work, he aimed to understand and hopefully to reduce the transmission of genetic disease in humans. His meeting with Patrick Steptoe, the details of which are re-examined, increased the significance for Edwards of infertility as an outcome of IVF. It also led to a creative long-term research partnership, initiated a long-term programme of public education in the UK about reproductive science and stimulated the development of bioethical thinking.

## Introduction

Robert G Edwards was awarded the 2010 Nobel Prize for Physiology or Medicine ‘for the development of in vitro fertilization’ (Nobel, 2010). There is a variety of accounts of the events leading up to this discovery and its acceptance, most of them by participants (see Johnson et al., 2010), but historical scholarship is rarer. This account uses verifiable sources to produce a historical narrative of the path to IVF that differs in a number of places from the conventionally accepted version and adds further detail.

## Materials and methods

Primary sources used were: the publications by Edwards and Steptoe during the 1950s, 1960s and 1970s; archives of the Royal Society of Medicine, Cambridge University, the Physiology Library at Cambridge and the personal papers of RG Edwards (courtesy of Ruth Edwards); unpublished transcripts of interviews with RG Edwards, K Elder and RL Gardner; personal recollections from the late 1970s by Edwards and Steptoe as recalled in interviews with Danny Abse for the autobiographical account ‘A Matter of Life’ and on film with Peter Williams; members of RG Edwards’ family and his colleagues and former students and staff members for clarificatory evidence about personal recollections by Edwards, for additional verifiable information and with whom to test some new interpretations.

## Results

### Childhood background

Robert Geoffrey Edwards was born on 27 September 1925 in the small Yorkshire mill town of Batley, the year of the Batley deluge and ‘great flood’. He arrived into a working-class family, and Edwards, who was known by his middle name of Geoff until he was 18, was the second of three brothers, with an older brother, Sammy and a younger, Harry. These brothers he describes as competitive, ‘all determined to win or, if not to win, to go down fighting’ (Edwards and Steptoe, 1980, p. 25). Sammy was named after his father, Samuel, who was frequently away from home working on the railways, maintaining the track in the Blea Moor tunnel on the Carlisle to Settle line. It was an unhealthy place to work, some 2600 m long and filled with coal-fired smoke that exacerbated Samuel’s bronchitis, a consequence of being gassed in World War I. The one perk of working on the railways was the free rail pass for the family’s annual holiday, which was regularly taken in far-away Southend-on-Sea, located near the mouth of the Thames and considered then to be a top-spot resort by working-class families.

Edwards’ mother, Margaret, was a machinist in a local mill. She came originally from Manchester, to where the family relocated when Edwards was about five, having been offered the relative security of a council house at 25 Highgate Crescent in the suburb of Gorton. It was in Manchester that Edwards was to receive his education. In those days, bright working-class children could take a scholarship exam at age 10 or 11 in competition for the few coveted places at a grammar school: the potential pathway out of poverty and even to University. All three brothers passed the exam, but Sammy decided against grammar school, preferring to leave education as soon as he could to earn. His mother was reportedly furious at this wasted opportunity, and so when her two younger sons passed, there was no question that they would continue in education and it was with that that Geoff/Bob progressed in 1937 to Manchester Central Boy’s High School (in the building that now houses Shena Simon College in Whitworth Street), which also claims James Chadwick FRS (1891–1974) as an earlier pupil. Chadwick, like Edwards, was a Cambridge professor and the 1935 Nobel Laureate in Physics for discovering the neutron (Massey and Feather, 1974). The Edwards’ summers were spent in the Yorkshire Dales, to where their mother took her sons to be closer to their father’s place of work. There, Edwards laboured on the farms and developed an enduring affection for the Dales.

These early experiences were formative. Edwards became a life-long egalitarian, for 5 years a labour party councillor (Ashwood-Smith, 2002), willing to listen to and talk with all and sundry, regardless of class, education, status and background. Second, he developed an enduring curiosity about agricultural and natural history and especially the reproductive patterns among the Dales’ sheep, pigs and cattle. Finally, he claimed great pride in being a ‘Yorkshire man’, traditionally having attributes of affability and generosity of spirit combined with no-nonsense blunt-speaking. Indeed, following his only meeting with Gregory Pincus (1903–1967; Ingle, 1971) at a conference in Venice in May 1966, at which Edwards, the young pretender, clashed with the ‘father of the pill’ over the timing of egg maturation in humans; he paid Pincus the biggest compliment he could imagine, saying ‘He would have made a fine Yorkshireman!’ (Edwards and Steptoe, 1980, p. 43).

**Figure 1:**
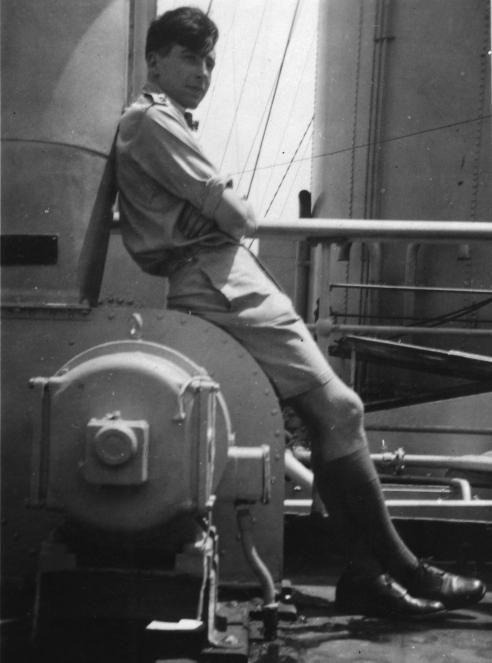
Edwards on National War Service 1940s (courtesy Ruth Edwards).

The intervention of World War II was to provide an unwelcomed interruption to Edwards’ education: when he left school in 1943, he was conscripted for war service into the British Army for almost 4 years (Figure 1). To his surprise, as someone from a working-class family, he was identified as potential officer material and sent on an officer-training course, before being commissioned in 1946. However, his army experiences were broadly negative, the alien lifestyle of the officers’ mess not being to his taste and reinforcing his socialist ideals. The one positive feature of his war service was the chance to travel overseas, particularly appreciated was his time in the middle-east. The years in the army were broken by 9 months compassionate leave back in the Yorkshire Dales, to which he was released to help and run a farm when his farmer friend there fell ill. So engaged did he become in farming life that, after discharge from the army in 1948, he returned home to Gorton, from where he applied to read agricultural sciences at the University College of North Wales at Bangor.

**Figure 2:**
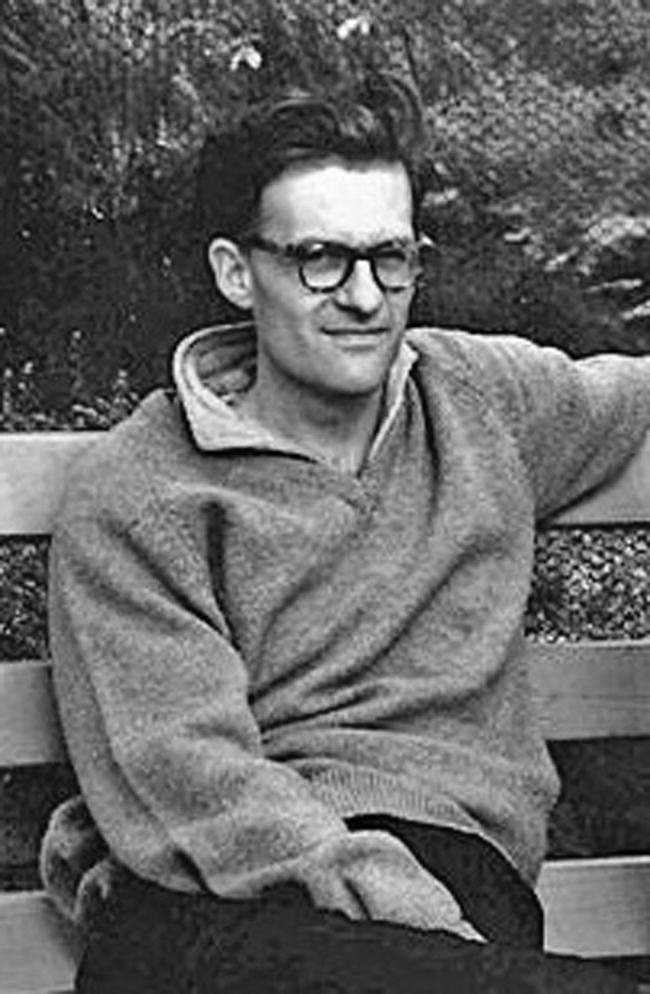
John Slee, 1960s (courtesy Ruth Edwards).

Having gained a place and a Government grant to fund it, the 6 or so months that intervened were occupied in a Government desk job in Salford, Greater Manchester, helping to organize the newly formed National Health Service. This office-work experience reinforced the anticipatory attractions of agricultural science. So his disappointment in the course offered at Bangor was acute. By that time, he was a relatively experienced 23-year-old, described by his impressionable 18-year-old public-school educated and self-described ‘unlikely’ friend, John Slee (Slee, 2002; Figure 2), as being ‘both ambitious and flexible, and unusually confident in his own judgement’. In Edwards’ confident judgement, the course on offer was not ‘scientific’, and he was bored through 2 tedious years of agricultural descriptions, after which he reported that his teachers were ‘glad to see the back of him’ in Zoology for a year, a course much more to his style and led by the more intellectually challenging Rogers Brambell FRS (1901–1970; Oakley, 1973). However, that year was not enough to salvage his honours degree, and in 1951, aged 26 he gained a simple pass. Unbeknown to him at the time, he was not alone in this undistinguished academic embarrassment, as neither ‘Tibby’ Marshall FRS (1878–1949; Parkes, 1950), the founder of the Reproductive Sciences, nor Sir Alan Parkes FRS (1900–1990; Polge, 2006), the first Professor of Reproductive Sciences at Cambridge, who was later to recruit Edwards there, distinguished themselves as undergraduates. In 1951, however, Edwards ‘was disconsolate. It was a disaster. My grants were spent and I was in debt. Unlike some of the students I had no rich parents ... I could not write home, “Dear Dad, please send me £100 as I did badly in the exams”’ (Edwards and Steptoe, 1980, p. 7).

However, his low spirits did not last long. He learnt that John Slee had been accepted on a postgraduate Diploma course in Animal Genetics at Edinburgh University under Conrad Waddington FRS (1905–1975; Robertson, 1977), who had moved there in 1947 from Christ’s College in Cambridge, home also to both Marshall and Parkes. Edwards applied, and, despite his pass degree and to his amazement, he was accepted. That summer, he worked in Yorkshire and Wiltshire harvesting hay, as well as portering bananas and heaving sacks of flour in Manchester docks and taking a menial job with a newspaper, all to earn enough to pay his way in Edinburgh (Edwards and Steptoe, 1980, p. 18).

### Family life

**Figure 3:**
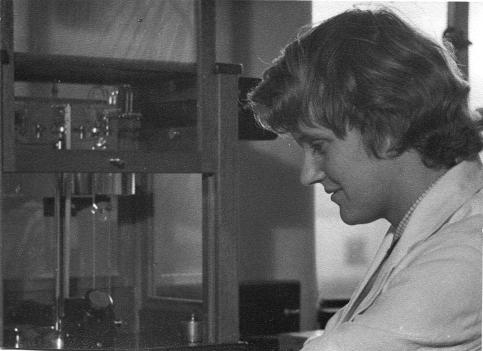
Ruth Fowler in laboratory, Edinburgh 1950s (courtesy Ruth Edwards).

In Edinburgh, Edwards not only started to map out his scientific career, but importantly also met Ruth Fowler (Figure 3), who was to become his life-long scientific collaborator and whom he was to marry in 1954, their five daughters following between 1959 and 1964: Caroline, Sarah, Jenny and twins Anna and Meg. When they met, according to Edwards, in a statistics class, Ruth was studying for a genetics degree. Edwards claims that he was initially somewhat overwhelmed, even ‘intimidated’ by Ruth’s august family background. Her father, Sir Ralph Fowler FRS (1889–1944; Milne, 1945) and her maternal grandfather, Lord Ernest Rutherford FRS (1871–1937; Eve and Chadwick, 1938), were not only both ‘titled’, but both also had the most impressive academic credentials imaginable: a world away from a working-class Northern family. Ralph Fowler was Plummer Professor of Mathematical Physics in Cambridge from 1932 to 1944. He was evidently an exceptionally talented mathematical physicist, a fine sportsman and ‘an inspirational teacher and leader of men’ (Milne, 1945). Back in Cambridge in 1919 after World War I, he was stimulated to work with Rutherford, who had recently arrived there to take the chair of Experimental Physics. Rutherford was the first Nobel laureate in Ruth’s family, having been awarded the 1908 Nobel prize for Chemistry ‘for his investigations into the disintegration of the elements, and the chemistry of radioactive substances’ (Eve and Chadwick, 1938).

Ralph Fowler not only worked under Rutherford, but in the course of doing so met his only daughter, Eileen, whom he married in 1921. They had four children, of whom Ruth was the last, born in December 1930. Tragically her mother died shortly afterwards and Ruth was to know only Mrs. Phyllida Cook as her ‘mother’, both families moving into Cromwell House in Trumpington, Cambridge and being brought up together (Milne, 1945). Her father, although himself unwell, was to undertake gruelling high security war work at the Ordnance Board and later at the Admiralty during World War II. His health deteriorated and he died at the relatively young age of 55 when Ruth was 13.

### Edwards, the research scientist

The intellectual spirit of scientific enquiry that Edwards experienced in Edinburgh fitted his aptitudes well, for Waddington rewarded his Diploma year with a 3-year PhD place (1952–55), followed by 2 years of post-doctoral research, and funded it to the princely sum of £240.00 per year (Edwards and Steptoe, 1980, p. 20). His chosen field of research was the developmental biology of the mouse. Edwards saw that to understand development involved engaging in an interdisciplinary mix, not just of embryology and reproduction, the conventional view at the time, but also of genetics. Given the scientific and social emphasis on genetics over the last 40 or so years, it is important to understand how advanced this view was in the 1950s, when genetic knowledge was still rudimentary and largely alien to the established developmental and reproductive biologists of the day, as Edwards himself was later to recall (Edwards, 2005). For example, it was in the 1950s that DNA was established as the molecular carrier of genetic information (Watson and Crick, 1953a,b; Franklin and Gosling, 1953; Wilkins et al., 1953), that it was first demonstrated that each cell of the body carried a full set of DNA/genes (Gurdon, 1962a,b; Gurdon et al., 1958) and that genes were selectively expressed as mRNA to generate different cell phenotypes (Weinberg, 2001). Moreover, it was only by the late 1950s that cytogenetic studies led to the accepted human karyotype as 46 chromosomes (Ford and Hamerton, 1956; Tjio and Levan, 1956), that agreement was reached on the Denver system of classification of human chromosomes (Denver Conference, 1960) and that the chromosomal aneuploidies underlying developmental anomalies such as Down, Turner and Klinefelter Syndromes were described (Ford et al., 1959a,b; Jacobs and Strong, 1959; Lejeune et al., 1959).

**Figure 4:**
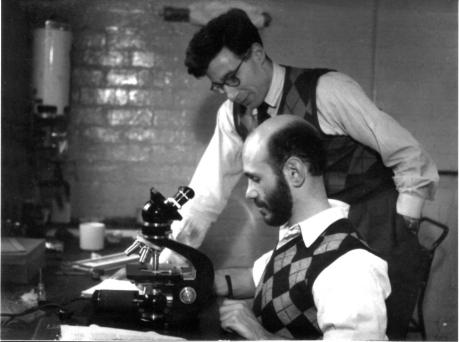
Julio Sirlin with Edwards, 1950s (courtesy Julio Sirlin).

The dates of these discoveries make Edwards’ research between 1952 and 1957 all the more remarkable. Working under his supervisor Alan Beatty, he generated haploid, triploid and aneuploid mouse embryos and studied their potential for development. In order to undertake what were, in effect, early attempts at ‘genetic engineering’ in mammals, he needed to be able to manipulate the chromosomal composition of eggs, spermatozoa and embryos. In mice, spermatozoa were abundant, and were studied in experiments mostly undertaken with a visiting Argentinian post-doc, Julio Sirlin (Figure 4), whom Edwards describes as being ‘... the first man with whom I collaborated who was prepared to work at my pace’ (Edwards and Steptoe, 1980, p. 27–28). Together they labelled spermatozoa radioactively *in vivo* in order to study the kinetics of spermatogenesis and then to follow the radioactive products post-fertilization, thereby to demonstrate the fate of the male contributions to early development. They also exposed males and/or their spermatozoa to various agents, such as chemical mutagens and UV- or X-ray irradiation, and examined the effects on sperm-fertilizing capacity, and where it was shown to be present, how the treatment impacted on development. In some cases, sperm activation of the egg was evident, but in the absence of any functional sperm chromatin, and so gynogenetic embryos were formed. These experiments resulted in 14 papers, including four in Nature, between 1954 and 1959 (see Gardner and Johnson, 2011, for a full bibliographic record for Edwards).

**Figure 5:**
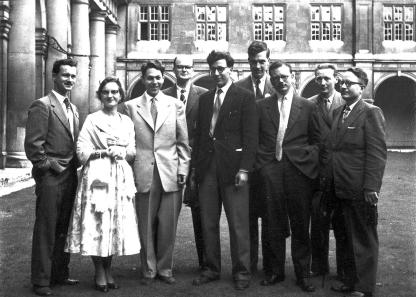
Edwards as ‘a very recent PhD student’ (centre) and Alan Gates (extreme left) at a meeting in Trinity College, Cambridge in the late 1950s (courtesy Ruth Edwards).

Eggs and embryos were not as abundant as spermatozoa, and overcoming this problem led Edwards to two discoveries that proved to be of particular significance for his later IVF work. First, working with his wife Ruth, they devised ways of increasing the numbers of synchronized eggs recoverable from adult female mice through a series of papers, the first published in 1957 (Fowler and Edwards, 1957), on the control of ovulation induced by use of exogenous hormones. In doing so, they overturned the conventional wisdom that superovulation of adults was not possible. Second, working with an American post-doc, Alan Gates (Figure 5; Edwards and Gates, 1959), Edwards described the remarkable timed sequence of egg chromosomal maturation events that led up to ovulation after injection of the ovulatory hormone, human chorionic gonadotrophin.

His 6 years in Edinburgh, between 1951 and 1957, give an early taste of his prodigious energy, resulting in 38 papers (Gardner and Johnson, 2011). Indeed so productive was this period that the last of the Edinburgh-based papers did not appear in print until 1963. These papers firmly placed the young Edwards at the forefront of studies on the genetic manipulation of development and started to attract attention.

It was also in Edinburgh that Edwards’ interest in ethics was first sparked by the interdisciplinary debates among scientists and theologians that Waddington organized, and, as a result, he went on what he describes as a ‘church crawl’, trying the 10 or so variants of Christianity on offer in 1950s Edinburgh. He did not emerge from his consumer testing ‘God-intoxicated’ (Edwards and Steptoe, 1980, p. 23–24), but convinced that man held his own future in his own hands. Edwards’ humanist ethical sympathies and antipathy to the ‘revealed truths’ of religion were to be developed further in all his later encounters (Gardner and Johnson, 2011).

### An American diversion

These 1950s’ studies in science and ethics were to form the platform on which Edwards’ later IVF work was to be based, but before that his interests and life took a diversion to the California Institute of Technology for the year 1957–1958. He describes his year at Caltech as being ‘a bit of a holiday’, but it was a holiday, which with hindsight had both distracting and significant consequences. He went there to work with Albert Tyler (1906–1968; Horowitz et al., 1969), an influential elder statesman of American reproductive science, working on spermatozoon–egg interactions. Caltech was then a hot bed of developmental biology, and Tyler had clustered around him an exciting group of young scientists, which included that year a visit by the English doyen of fertilization, Lord Victor Rothschild FRS (1910–1990; Reeve, 1994). Rothschild was later to clash scientifically with Edwards over his IVF work (Rothschild, 1969), a clash in which the younger man triumphed again (Edwards et al., 1969b), just as he had with Pincus. Tyler was exploring the molecular specificity of egg–spermatozoon interactions and had turned for a model to immunology. Immunology was then at an exciting phase in its development, with the engaging Sir Peter Medawar FRS (1915–1987, Nobel Laureate in Physiology or Medicine, 1960; Mitchison, 1990), influentially for Bob, extending his ideas on immunological tolerance to the paradox of the ‘fetus as an allograft’: a semi-paternal graft nonetheless somehow protected from maternal immune attack inside the mother’s uterus (Medawar, 1953). This confluence of reproduction and immunology excited Edwards’ restless curiosity and hence the choice of Tyler. Significantly, the subject also offered funding possibilities via the Ford and Rockefeller Foundations and the Population Council, which were increasingly concerned about world population growth and the need for better methods to control fertility (Clarke, 1998, pp. 207–230; Connelly, 2008; Marks, 2001, p. 31, pp. 195–236). Immuno-contraception then seemed to offer tantalizingly specific possibilities, alas not much closer to being realized today (Naz et al., 2005).

**Figure 6:**
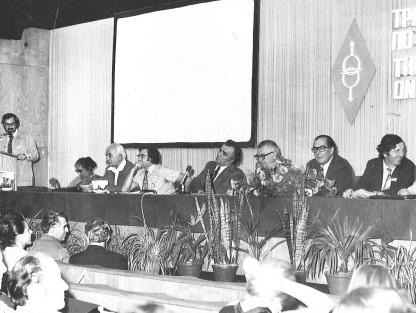
Edwards at one of the Varna meetings on Immuno-Reproduction; Schulman is speaking and to Edwards left is Bratanov, and seated two to his right is Shanta Rao (courtesy Barbara Rankin).

So when Edwards returned to the UK from CalTech in 1958 at Alan Parkes’ invitation to join him at the Medical Research Council (MRC) National Institute for Medical Research (NIMR) at Mill Hill in north London, it was to work on the science of immuno-contraception (Ashwood-Smith, 2002). This period in the USA initiated a series of 23 papers on the immunology of reproduction between 1960 and 1976 (Gardner and Johnson, 2011). It also prompted Edwards’ first involvement in founding an international society in 1967 in Varna Bulgaria, (Figure 6) when the International Coordinating Committee for the Immunology of Reproduction was created (Rukavina, 2008). Immuno-reproduction was, in retrospect, to prove a distracting diversion from what was to become Edwards’ main work, albeit one that continued to enthuse and stimulate his imagination for many years. Indeed, it was his research into immuno-reproduction that led serendipitously to his first meeting with Patrick Steptoe (see later). The period at Mill Hill, between 1958 and 1962, seems to have been a period of increasing intellectual conflict for him. Whilst enthusiastic about the science underlying immuno-contraception, his old interests in eggs, fertilization and, in particular, the genetics of development were gradually reasserting themselves. His day job was therefore increasingly supplemented by evening and weekend flirtations with egg maturation.

### The crucial egg-maturation studies

The stimulus that re-awakened Edwards’ interest in eggs was provided by the then recent consensus about the number of human chromosomes and, more particularly the descriptions in 1959 of the pathologies in man that resulted from chromosomal anomalies. Thus, his 1962 Nature paper begins: ‘Many of the chromosomal anomalies in man and animals arise through non-disjunction or lagging chromosomes during meiosis in the oocyte. Investigation of the origin and primary incidence of such anomalies would be greatly facilitated if meiotic stages etc., were easily available’ (Edwards, 1962).

The idea that these aneuploidies in humans might result from errors in the complex chromosomal dance that he and Gates had observed in maturing mouse eggs drove his thinking. The possible clinical relevance of his work on egg maturation and aneuploidy in the mouse was becoming significant.

So Edwards resumed his experimenting with mice, trying to mimic *in vitro* the in-vivo maturation of eggs, one rationale being that this route would open the possibility of similar studies in humans, in which not even induced ovulation had then been described (Gemzell, 1962). He tried releasing the immature eggs from their ovarian follicles into culture medium containing the ovulatory hormone human chorionic gonadotrophin, to explore whether he could simulate their in-vivo development. Amazingly he found it worked first time, the eggs seemed to mature at the same rate as they had *in vivo*. However, they did so whether or not the hormone had been added. The eggs evidently were maturing spontaneously when released from their follicles. The same happened in rats and hamsters. If this also were to happen in humans, then the study of the chromosomal dance during human egg maturation was a realistic practical possibility, as was IVF and thereby studies on the genetics of early human development. Edwards’ excitement at seeing eggs spontaneously maturing was temporarily blunted by his library discovery that Pincus in the 1930s (Pincus and Enzmann, 1935; Pincus and Saunders, 1939) and MC Chang (1908–1991; Greep, 2010; Chang, 1955) earlier in the 1950s had been there before him, using both rabbit and, Pincus claimed, human eggs.

In order to pursue his cytogenetic studies on maturation, he needed a reliable supply of human ovarian tissue from which to retrieve and mature eggs. This requirement posed difficulties for a scientist with no medical qualification, given the elitist attitudes and lack of scientific awareness then prevalent amongst most of the UK gynaecological profession (Johnson et al., 2010; MRC, 1969; RCOG, 1967). His first breakthrough came with Molly Rose, who was a gynaecologist at the Edgeware General Hospital, northwest London, near Mill Hill. Edwards was introduced to her through John Humphrey FRS (1915–1997; Askonas, 1990), who was the medically qualified Head of Immunology at Mill Hill. Humphrey, notwithstanding his more privileged social background, was a kindred spirit for Edwards, sharing his passion for science, its social application and utility, as well as his left-wing politics; indeed he had been a Marxist until 1940 and was for many years denied entry to the USA as a result. Edwards asked Humphrey if he knew anyone who might be helpful, and he not only suggested Rose, but also offered to arrange an introduction. Rose was to provide biopsied ovarian samples intermittently for the next 10 years.

Between 1960 and 1962, Edwards used human ovarian biopsies provided by Rose to try to repeat and extend Pincus’ observations from the 1930s. Given the sporadic supply of human material, he also tried dog, monkey and baboon ovarian eggs, but in all cases with limited success compared with smaller rodents. In the 1962 Nature paper (Edwards, 1962), he cautiously interprets the few maturing human (3/67), monkey (10/56) and baboon (13/90) eggs that he had observed as most likely arising from in-vivo stimulation and thus partially matured at the time of their recovery from the biopsy. He suggests that Pincus’ observations on human eggs are also likely to be artefactual, the source of his Venice spat with Pincus some 4 years later (vide supra). This 1962 paper ends with the report of an ingenious experimental approach to try and persuade the reluctant human eggs to mature. Thus, the ovarian arteries of patients undergoing ovarian removal were cannulated and perfused with hormones post-removal, perhaps unsurprisingly in retrospect, without success.

However, by this time, his quest for human eggs, and his dreams of IVF and studying the genetics and development of early human embryos, had reached the ears of the then Director of the Institute, Sir Charles Harington FRS (1897–1972; Himsworth and Pitt-Rivers, 1972), who banned any work on human IVF at NIMR (Edwards and Steptoe, 1980, p. 48). Alan Parkes was no longer able to defend Edwards, having left in 1961 to take up his chair in Cambridge and, although he had asked Edwards to join him there, funding was not available until 1963. So by the time Edwards left Mill Hill in 1962 for a year in Glasgow, he had encountered a taste of the opposition to come.

### Glasgow and stem cells

Edwards had accepted an invitation from John Paul to spend a year in the Biochemistry Department at Glasgow University. Paul was then the acknowledged master of tissue culture in the UK and had got wind of some experiments that Edwards had been doing on the side at NIMR attempting to generate stem cells from rabbit embryo cultures (Edwards, 2005). The objective of this strategy was to use these stem cells to study early developmental mechanisms, either *in vitro*, or *in vivo* after their incorporation into embryos. Paul had proposed that they work together, with fellow Glasgow biochemist Robin Cole, to see what progress might be made. This must have been an attractive invitation, not simply because the challenge was scientifically interesting, but also because Edwards could learn more about culture media for his eggs and hopefully later embryos, then an uncertain prospect, successful mouse embryo culture only recently having been described (McLaren and Biggers, 1958). However, by this time, the Edwards family was growing, so Ruth remained in north London with their young daughters, while her husband commuted to Glasgow for the working week.

The collaboration was to result in two papers (Cole et al., 1965, 1966) remarkable for their prescience. They describe the production of embryonic stem cells from both rabbit blastocysts and the inner cell masses dissected from them. The cells were capable of proliferating through over 100 generations and of differentiating into various cell types. These experiments were initiated some 20 years before Evans and Kaufman (1981) described the derivation of embryonic stem cells from mice. That this work has largely been ignored by those in the stem cell field is probably mainly attributable to its being too far ahead of its time (Edwards, 2001). Thus, reliable molecular markers for different types of cells were not available then, nor were appropriate techniques with which to critically test the developmental potential of the cultured cells.

### The move to Cambridge

Edwards arrived in Cambridge from Glasgow in 1963 as a Ford Foundation Research Fellow. He had previously visited Cambridge at least once, as ‘a recently graduated PhD’ in the late 1950s for a conference on Reproduction held in Trinity College (Figure 5), where he recalls meeting some of the big names in the subject, including John Hammond, Alan Parkes, MC Chang, Thaddeus Mann, Rene Moricard, Bunny Austin and Charles Thibault (Edwards, 2005). Although Edwards was to remain in Cambridge for the rest of his career, in 1963 his reactions to the place were mixed. He describes how he immediately reacted against the then extant ‘misogynist public-school traditions; the exclusivity’, ‘the privileges given to the already privileged’. But he set against that the ‘sheer beauty of the place’, the concern with the truth and high seriousness’, the ambience of scientific excellence ... I was surrounded by so many talented young men and women’ (Edwards and Steptoe, 1980, p. 51). He, Ruth and his five daughters settled into a house in Gough Way, off the Barton Road.

**Figure 7:**
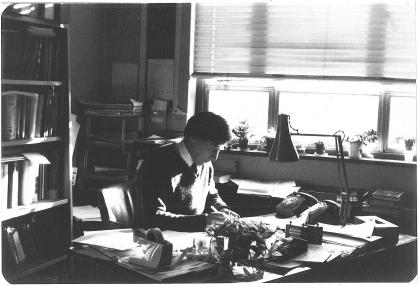
Edwards in his office backing onto Downing Place in the Marshall laboratory (1970s) (courtesy Barbara Rankin).

Edwards worked in a cluster of seven smallish rooms at the top of the Physiological Laboratory backing onto Downing Place (Figure 7). These were known collectively as the ‘Marshall laboratory’ and were to be shared eventually with two other groups. One group was led initially by Sir Alan Parkes, the first Mary Marshall and Arthur Walton Professor of Reproductive Physiology at the University (Polge, 2006), who had arrived in 1961. His group included scientists with mainly zoological or comparative interests, such as his wife Ruth Deansley, Bunny Austin and Dick Laws FRS, who with Parkes was often away ‘in the field’ collecting material, especially in Uganda at the Nuffield Unit of Tropical Animal Ecology (Polge, 2006). Much of this material was examined histologically under the skilled eye of Frank Lemon, senior technician. Research students included Martin Richards, CJ Dominic, Margaret Mitchell and Barbara Weir (Parkes, 1985, pp. 77–83). Parkes was also much involved at this time in writing and committee work, especially with the World Health Organization which was then becoming concerned about world population growth and ways to curb it (Polge, 2006). Parkes was also acting as unpaid company secretary to the then fledgling Journal of Reproduction and Fertility (called Reproduction since 2001; Parkes, 1985, pp. 332–334; Cook, 1994; Clarke, 2007).

**Figure 8:**
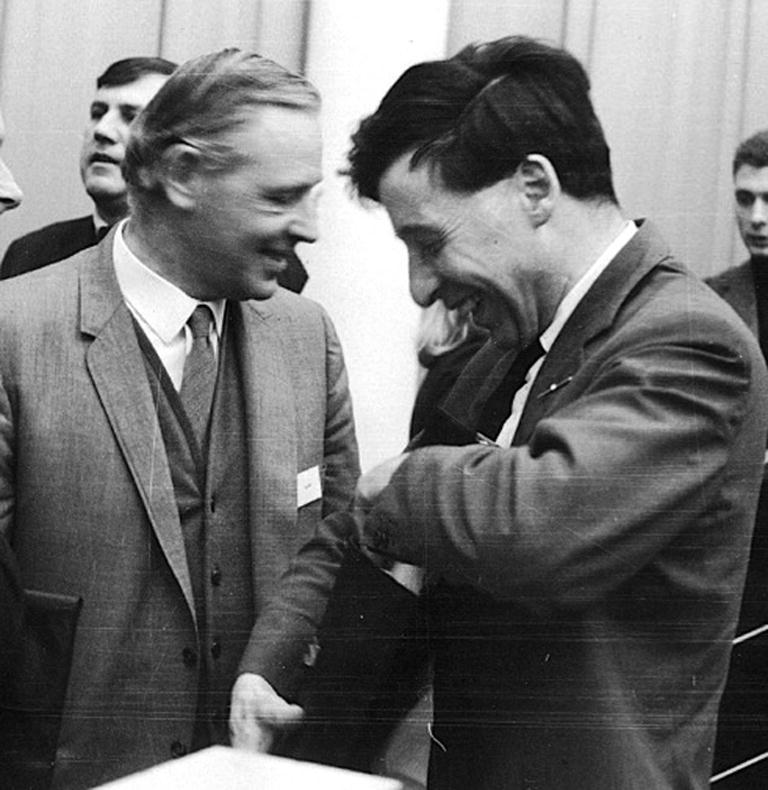
Edwards with ‘Bunny’ Austin (1960s) (courtesy Ruth Edwards).

**Figure 9:**
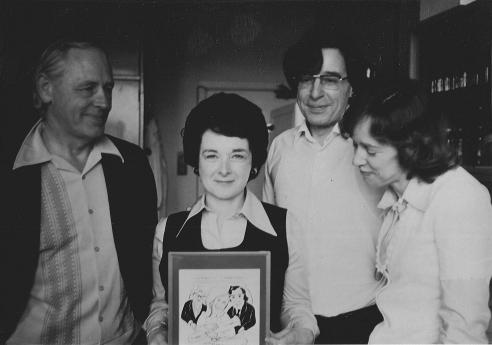
Barbara Rankin holding a cartoon of Edwards, Steptoe and Purdy holding Lousie Brown, drawn by Alan Handyside, who also took the photograph. With Austin (left), Edwards and Purdy (right), after the return of the latter two from Oldham and the birth of Louise Brown in 1978 (courtesy Barbara Rankin).

In 1967, Parkes retired. Edwards applied for his chair on 6 January 1966 (Edwards, 1966), but was unsuccessful, the chair passing to Thaddeus Mann FRS (1908–1993; Polge, 1993), who worked on the biochemistry of semen. Mann decided not to relocate to the Physiology Laboratory from his Cambridge base at the Agricultural Research Council Unit of Reproductive Physiology and Biochemistry at Huntingdon Road, where he was Director. Neither was the leadership of the Marshall laboratory to pass to Edwards, as the University appointed as its head his more senior colleague and friend Colin ‘Bunny’ Austin (1914–2004; Short, 2004), who had been in Cambridge intermittently since 1962 (Figures 8 and 9). Austin was elected the first Charles Darwin Professor of Animal Embryology (1967–1981) and began attracting several upcoming reproductive biologists to the Marshall Laboratory, including John Marston, David Whittingham and Matthew Kaufmann. In addition, a new group was formed in 1967, with the arrival from the Strangeways laboratory of Denis New (1929–2010), as University lecturer in Histology (Arechaga, 1997). New built a group comprising initially research assistant Pat Coppola (to be followed later by Stephanie Ellington) and PhD students Chris Steele and David Cockroft, later joined by post-doc Frank Webb, and visiting scientists such as Joe Daniels Jr, on leave from the University of Colorado.

**Figure 10:**
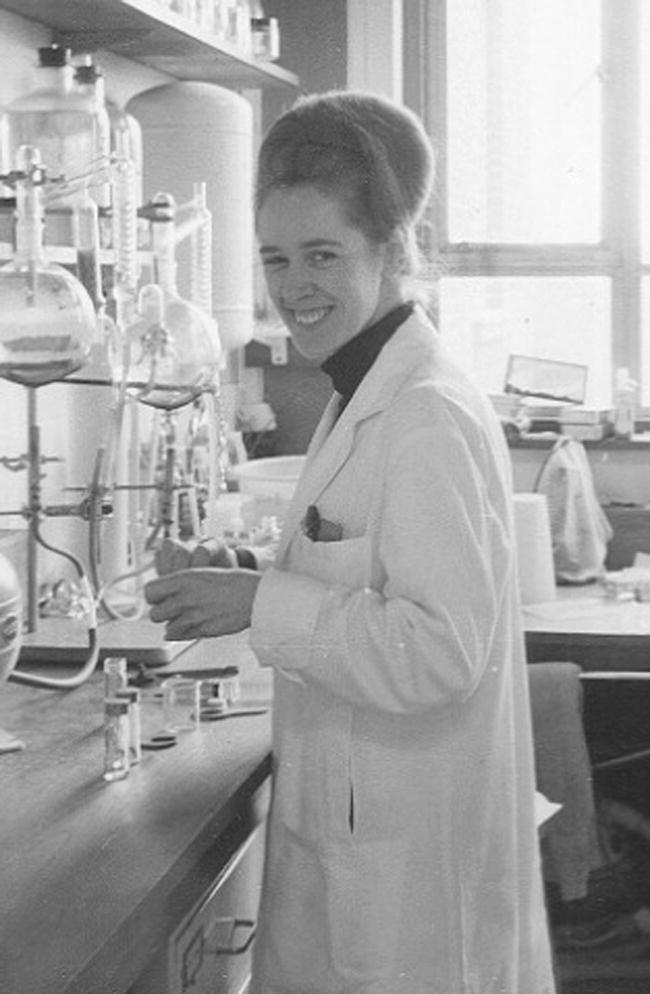
Jean Purdy (1946–1985) (courtesy Barbara Rankin).

It was against this varied scientfic background that Edwards, who was already 38 when he arrived in Cambridge, began for the first time to assemble his own group. Initially, his technical assistance was provided by Clare Jackson and then Valerie Hunn, after whom he recruited Jean Purdy (Figure 10) in 1968, one of her attractions being her nursing qualification, a sign of the increasing importance that his forays into use of clinical material was assuming. Purdy was to stay with him until her early death aged 39 in 1985 (Edwards and Steptoe, 1985). Also joining him as part-time secretary in 1969, Barbara Rankin (b. 1933; Figure 9), was to remain with him until 1987. He also began recruiting his first graduate students. Initially, he helped co-supervise (with Alan Parkes) Anne Vickers (1967), who sexed fixed whole-mouse blastocysts by karyotyping. This work led directly to his collaboration on preimplantation genetic diagnosis (PGD) (see later) with Richard Gardner, one half of his first pair of graduate students, the other being this author. The two students started PhD training with Edwards in 1966 (Gardner, 2011; Gardner and Johnson, 1991, 2011). Gardner studied early mouse embryology from 1966 to 1971, and until 1973 as a post-doctoral worker, before moving to Zoology in Oxford. This author worked on immuno-reproduction from 1966 to 1969, returning as a post-doc between 1971 and 1974 after 2 years in the USA before moving to the Anatomy Department in Cambridge.

From 1969 onwards, Edwards’ group increased in size substantially as more accommodation was made available to the Marshall laboratory. David Griffin (now retired from the World Health Organization) was to join as Head Technician between 1970 and 1975, with junior technicians including Sheila Barton, Sally Fawcitt, Sylvia Jackson, Vinitha Dharawardena and Brenda Dickstein, in addition to Jean Purdy. Early graduate students recruited included Roger Gosden (1970–1974), Carol Readhead (1972–1976) and Rob Gore-Langton (1973–1978), all working on follicle growth, Craig Howe (1971–1974) working on immuno-reproduction and Azim Surani (1975–1979) working on implantation. A ‘third generation’ of graduate students also arrived, for example, Janet Rossant (from 1972) studied with Gardner and Alan Handyside (from 1974) studied with Johnson. Post-doctoral workers also arrived, including Ginny Papaioannou (1971–1974), Hester Pratt (1972–1974) and Frank Webb (1976–1977). Ruth Fowler-Edwards also resumed working in the laboratory, developing hormonal assays and studying the endocrine aspects of follicle development and early pregnancy. Thus, slowly until 1969, and more rapidly thereafter, Edwards built a lively group, its members working in diverse areas of reproductive science that reflected his own broad interests and knowledge. Moreover, Edwards encouraged a spirit of open communication and egalitarianism, which extended across all three groups, with sharing of resources, space, equipment, knowledge and ideas, as well as social activities.

Through the 1960s, Edwards was funded by the Ford Foundation via grants first to Parkes and then to Austin to continue work on basic reproductive mechanisms, with an eye to developing new methods of fertility control. So he continued to pursue both the immunology of reproduction and egg maturation, for the latter collecting pig, cow, sheep, the odd monkey and some human eggs. He showed that eggs of all these species would indeed mature *in vitro*, but that the eggs of larger animals simply needed longer than those of smaller ones, human eggs taking up to 36 h rather than the 12 h or less erroneously reported by Pincus. These cytogenetic studies were reported in two seminal papers in 1965 (Edwards, 1965a,b), both of which are primarily concerned with understanding the kinetics of the meiotic chromosomal events during egg maturation. In its discussion, the Lancet paper displays a breathtaking clarity of vision as Edwards sets out a programme of research that predicted the events of the next 20 years and beyond (Table 1). Significantly, if not surprisingly given his research interests, the early study and detection of genetic disease is afforded a heavy focus compared with the slight emphasis on infertility alleviation.

**Figure 11:**
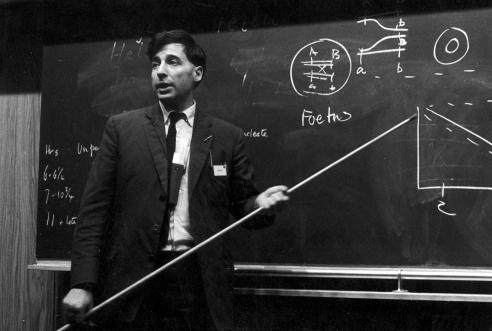
Edwards talks about his ‘production line’ hypothesis (late 1960s) (courtesy Ruth Edwards).

This genetic focus continues in his research papers over the next 4 years. Thus, within 3 years, working with graduate student Gardner, he provided proof of principle for PGD, in a paper on rabbit embryo sexing published in 1968 (Gardner and Edwards, 1968), a paper that was to anticipate the development of PGD clinically by some 22 years (Theodosiou and Johnson, 2011). Likewise, working with Cambridge geneticist Alan Henderson, Edwards was to develop his ‘production line theory’ of egg production to explain the origins of maternal aneuploidy in older women. Thus, the earliest eggs to enter meiosis in the fetal ovary were shown to have more chiasmata and to be ovulated earlier in adult life than the those entering meiosis later in fetal life (Figure 11; Edwards, 1970; Henderson and Edwards, 1968).

### The problem of fertilization of the human egg

Notwithstanding his broad range of scientific interests, Edwards’ ambitions to achieve IVF in humans remained undiminished. In 1966, this was no trivial task, having been accomplished convincingly only in rabbit and hamster (Chang, 1959; Yanagimachi and Chang, 1963). In trying to achieve this aim, he was engaging in two struggles: the first being simply but critically the continuing practical difficulty in obtaining a regular supply of human ovarian tissue. Local Cambridge sources proved unreliable and Rose was now 2–3 h drive away in London, so during the summer of 1965, Edwards turned to the USA for help and approached Victor McKusick, a leading American cytogeneticist at The Johns Hopkins University. There he initiated his longstanding contact with Howard and Georgeanna Jones in Obstetrics and Gynecology (Jones, 2002). The supply of American eggs they generated during his 6-week stay allowed him to confirm the maturation timings that were published in 1965.

However, it was the second scientific struggle that was then occupying most of his attention, namely that in order to fertilize these in-vitro matured eggs, he had to ‘capacitate’ the spermatozoa, a final maturation process which spermatozoa undergo physiologically in the uterus and that is essential for the acquisition of fertilizing competence. Failing to achieve this convincingly at Johns Hopkins, he made a second transatlantic summer journey in 1966 to visit Luther Talbot and his colleagues at Chapel Hill. He tried a variety of ways (Edwards et al., 1966) to overcome the problem of ‘sperm capacitation’, one of the most ingenious of which was to construct a 2.5-cm-long chamber from a nylon tube, plugged at each end, and with holes drilled in the walls which were encased in panels made of Millipore membrane (Edwards et al., 1968). The chamber, which had a short thread attached to it, fitted snugly inside an intrauterine device’s inserter tube and so could be placed into the volunteer woman’s uterus intra-cervically at mid-cycle, where it sat for up to 11 h before being recovered by gently pulling on the thread, exactly as was being done routinely for insertion and removal of intrauterine devices. By placing spermatozoa within the chamber, the membrane of which permitted equilibration of its contents with uterine fluid, he hoped to expose them to a capacitating environment. However, this ingenious approach, like the many others, failed, in this case most probably because the chamber itself induced an inflammatory response or a local bleed. For all the ingenuity of his various experimental approaches to achieve capacitation, and despite the occasional evidence of early stages of fertilization using such spermatozoa, no reliable evidence for the completion of the process was forthcoming. Then in 1968 both struggles began to resolve.

### The meeting with Patrick Steptoe

**Figure 12:**
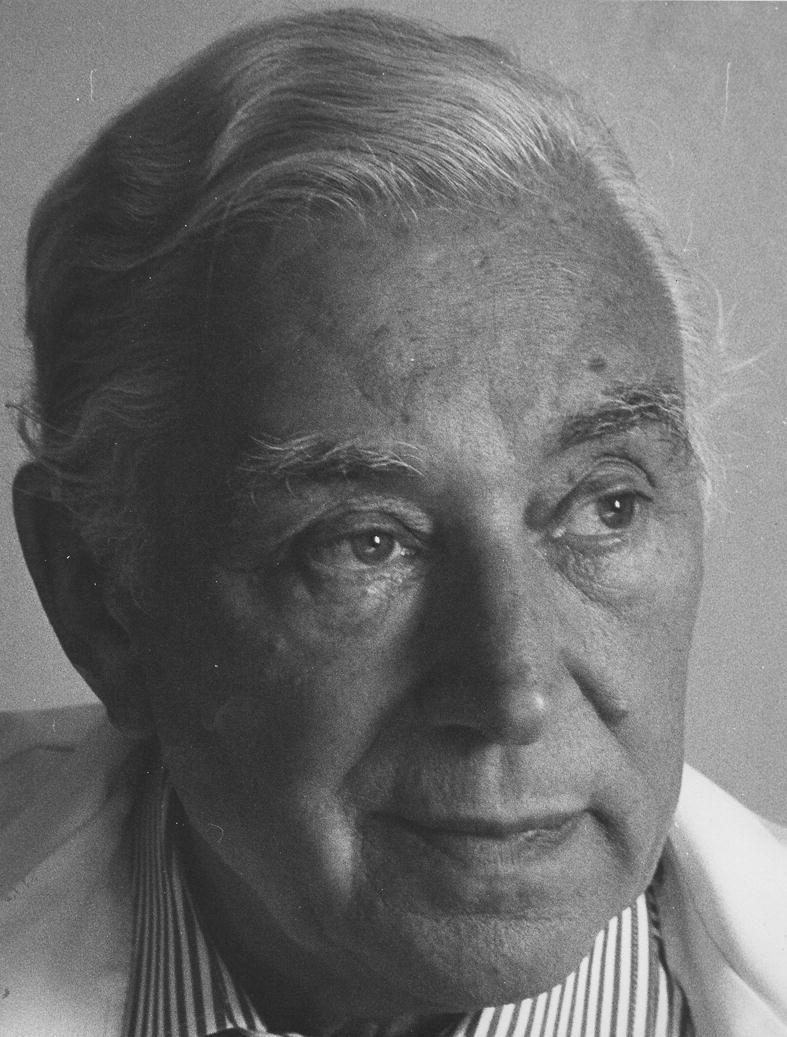
Patrick Steptoe (1913–1988) (courtesy Andrew Steptoe).

Patrick Steptoe (1913–1988; Figure 12) had been Consultant Obstetrician at Oldham General Hospital since 1951 (Edwards, 1996), where for several years he had been pioneering the development and use of the laparoscope in gynaecological surgery (Edwards, 1996; Steptoe, 1967a). Much to his frustration, his progress had fallen on the largely deaf ears of the conservative gynaecological hierarchy, and indeed incited considerable opposition and some outright hostility (Philipp, 1988; Edwards, 1996). Edwards’ claims that he was scanning the medical and scientific journals in the library and came across a paper by Steptoe describing his experiences with laparoscopy (Edwards and Steptoe, 1980, p. 59; Edwards, 1989, 1996). Edwards goes on to describe how he rang Steptoe to discuss a possible collaboration, but was ‘warned off’ Steptoe by London gynaecological colleagues (Edwards, 1978). This warning, and the daunting prospect of a collaboration in far-away Oldham, deterred him from following through. Edwards reports finally actually meeting Steptoe later at a meeting at the Royal Society of Medicine, at which, ironically, Edwards was talking about his work on immuno-reproduction, not his attempts at IVF.

The Steptoe paper that Edwards found that day in the library was cited in his tributes to the then deceased Steptoe (Edwards, 1989, 1996) as being a Lancet paper entitled ‘Laparoscopy and ovulation’ (Steptoe, 1968). However, these later recollections do not withstand scrutiny. Thus, the Lancet paper cited was published in October 1968, but their first meeting was in fact earlier that year, on Wednesday 28 February 1968 at a joint meeting of the Section of Endocrinology of the Royal Society of Medicine with the Society for the Study of Fertility held at 1 Wimpole Street (Table 2; Hunting, 2002, pp. 252–253). Moreover, according to Steptoe (1969), they had already commenced collaborating prior to October 1968; indeed their first paper together was submitted for publication later that year in December 1968 (see next section). Clearly, the paper read by Edwards must have been another, earlier than October 1968, one that proceeded February 1968 by several months. Indeed, in an earlier account, Edwards describes the library ‘Eureka’ moment as occurring in ‘one autumn day in 1967’ (Edwards and Steptoe, 1980, p. 59). So which was the paper by Steptoe that Edwards saw and what about it attracted his attention? Looking at Steptoe’s possible publications in journals, there is none listed for 1967, but there are two 1967 conference reports and Steptoe’s book on gynaecological laparoscopy (Steptoe, 1967a–c). Of the few journal papers, only two concern laparoscopy, one from January 1966 in the British Medical Journal (BMJ), and one from August 1965 in the Journal of Obstetrics and Gynaecology of the British Commonwealth (JOGBrC). Which of these five publications (Table 3) did Edwards read?

The 1966 BMJ publication (Steptoe, 1966) is a letter headed ‘The fifth freedom’. It responds to a paper by Sir Dugald Baird on the ‘problem of excessive fertility in women’. Steptoe concurs that there is a problem, but disagrees with the proposed contraceptive solution, advising laparosopic sterilization for women as safer and more effective. He also discusses how laparoscopy can be used post-operatively to confirm that tubes were indeed blocked. The JOGBrC paper (Steptoe, 1965) is titled ‘Gynaecological endoscopy – laparoscopy and culdoscopy’, and reviews the history of endoscopy and Steptoe’s experiences with it. It is in essence a very abbreviated version of his book (Steptoe, 1967a), which was to be published in the following year. The two reports of the conference proceedings (Steptoe, 1967b,c) are slightly more detailed accounts than the BMJ letter and are much abbreviated versions of the JOGBrC paper.

Of these five publications, the Sydney conference proceedings (Steptoe, 1967c) can probably be dismissed as they only arrived in a Cambridge library in May 1968. The proceedings from Stockholm (Steptoe, 1967b) are now no longer available in Cambridge, but evidence of their presence in the Physiology library in 1967 has been uncovered in an old catalogue record, and so they would have been newly available to Edwards from November 1967 at the earliest. The BMJ letter (Steptoe, 1966) seems unlikely. Although Edwards was involved in contraceptive studies over this period through his work on immuno-reproduction and was a member of the Royal Society Population Study Group at the time (Polge, 2006, p. 280), and so may well have read this correspondence, it seems unlikely that the BMJ letter would have caught his eye to such dramatic effect and so long after its publication in January 1966, being readily available each week in the Physiology Library. The JOGBrC paper from 1965 was located in the University library, which was physically more remote from Physiology and not so immediately available to Edwards. However, it was in exactly the sort of clinical journal that he might then have been trawling retrospectively in his attempts to solve the problem of sperm capacitation. Thus, whereas in the more recent accounts of these events, Edwards (1989, 1996) records his motivation for contacting Steptoe as being the potential value of the laparoscopic approach for egg collection, in 1967 eggs were not foremost on his mind. Indeed in two earlier accounts, one written (Edwards and Steptoe, 1980, p. 59) and one spoken (Edwards, 1978; Supplementary video), Edwards claims he saw laparoscopy as a way of recovering capacitated spermatozoa from the oviduct by flushing with a small volume of medium: ‘a practical way ... of letting spermatozoa be in contact with the secretions of the female tract’ (Edwards and Steptoe, 1980, p. 59). He says he actually rang Steptoe to ask whether this really was possible and was reassured by him that this was the case. However, the only publication by Steptoe that explicitly lays out this possibility is his book (Steptoe, 1967a). Thus, on page 27 he reports ‘By means of laparoscopy, Sjovall (1964) has carried out extended post-coital tests and has recovered spermatozoa from the fimbriated end of the tubes ...’; and on page 70, he writes ‘An extended post-coital test can be done by aspirating fluid from the tubal ostium ...’. Moreover, Steptoe’s book arrived in the University library in March 1967. However, against this conclusion sits Steptoe’s recollection that Edwards had rung him just before his book was published, that is before March 1967 (Edwards and Steptoe, 1980, pp. 75–76). This memory conflicts with both his and Edwards’ memories elsewhere of the phone conversation being in the autumn of 1967, so the matter remains one for conjecture, but the book seems the most likely source. It is possible that Edwards’ attention was drawn to the book by a review of it in the BMJ on 11 November 1967 (Morrison, 1967).

### The fertilization of the human egg resolved

Despite the initiation of the collaboration with Steptoe, the actual solution to the capacitation problem lay nearer to home than Oldham: in the laboratories shared with Austin. In the early 1950s, Austin, and independently MC Chang, had discovered the requirement for sperm capacitation (Austin, 1951; Chang, 1951). After his appointment to the Cambridge chair, Austin’s first graduate student (1967–1972) was Barry Bavister, who set to work to try and resolve the factors influencing the capacitation of hamster spermatozoa in vitro. In 1968, Bavister discovered a key role for pH, showing how higher rates of fertilization could be obtained by simply increasing the alkalinity of the medium (Bavister, 1969). Edwards seized on this observation and co-opted Bavister to his project. That proved to do the trick, and in December 1968 Edwards, together with Bavister and Steptoe, submitted the paper to Nature, in which IVF in humans is described convincingly for the first time (Edwards et al., 1969a).

The 1969 Nature paper makes modest claims. Only 18 of 56 eggs assigned to the experimental group showed evidence of ‘fertilization in progress’, only two of which are described as having the two pronuclei to be expected if fertilization was occurring normally (Table 4). However, like Edwards’ other papers, this one is a model of clarity, describing well-controlled experiments, cautiously interpreted. Despite the relatively small numbers, this paper convinced where previous claims had failed (Hayashi, 1963; Petrov, 1958; Petrucci, 1961; Rock and Menkin, 1944; Shettles, 1955, pp. 505–510; Yang, 1963), precisely because the skilled hands and creative intellect that lay behind it are so evident from its text.

The provenance of the eggs described in the 1969 paper is not immediately clear from the paper itself. All were obtained by in-vitro maturation after ovarian biopsy. In addition to Steptoe’s co-authorship, four other gynaecologists are thanked in the Acknowledgements section of the paper: Molly Rose, Norman Morris (1920–2008; Professor of Obstetrics and Gynaecology at Charing Cross Hospital, London from 1958 to 1985; Anon, 2008), Janet Bottomley (1915–1995; Consultant Obstetrician and Gynaecologist at Addenbrooke’s Hospital, Cambridge from 1958 to 1976) and Sanford Markham (b. 1934; Chief of the Section of Obstetrics and Gynaecology at the US Air Force Hospital, South Ruislip, to the north west of London from 1967 to 1972). Markham, now Professor of Obstetrics and Gynecology at Florida International University, Miami, writes:

“I believe that I met Bob by introduction from Dr. Roger Short at a Royal College of Medicine conference in London ... probably in early 1968 [possibly the Royal Society of Medicine’s 28 February meeting at which Steptoe and Edwards first met?]. Bob mentioned that he was in need of ovarian tissue from reproductive aged women ... I offered to obtain tissue if we could work out a scheme to transport the tissue ... to Cambridge ... He provided the media and container and a driver that came to our hospital ... I remember three samples, however, there may have been others. In each case the whole wedge ... or ... ovary was sent (after sampling for pathology). In all cases the patients provided their consent for utilization of their tissue for research. They were not told what the research work involved. These samples were most likely sent in mid to late 1968 and possibly in early 1969 ... These ... were planned surgeries which were accomplished at specific times in their menstrual cycle. Unfortunately, I do not know if the tissues supplied were indeed the tissues used in data for his 1969 paper published in Nature.”

It is possible that they were used, but unsuccessfully, not contributing to the 18 eggs showing fertilization. Thus, according to Edwards (Edwards and Steptoe, 1980, pp. 81–82), Jean Purdy drove to Edgeware General Hospital to collect:

“the last piece of ovarian tissue that I was to obtain from the Edgware General Hospital. It yielded me 12 human eggs. Those eggs were soon ripening in mixtures of culture medium I had used over many years to which some of Barry [Bavister]’s fluid had been added. Thirty-six hours later we judged that they were ready for fertilization.”

Nine of these were inseminated, leaving three as controls. Ten hours later, when Edwards and Bavister returned to the laboratory late at night:

“A spermatozoon was just passing into the first egg ... An hour later we looked at the second egg. Yes, there it was, the earliest stages of fertilization. A spermatozoon had entered the egg without any doubt – we had done it ... We examined other eggs and found more and more evidence. Some ova were in the early stages of fertilization with the sperm tails following the sperm heads into the depths of the egg; others were even more advanced with two nuclei – one from the sperm and one from the egg – as each gamete donated its genetic component to the embryo.”

This (unverified) account suggests that Rose provided the first group of eggs to be fertilized in ‘Bavister’s medium’. Moreover, since only 18 of the eggs in the paper showed evidence of fertilization (Table 4), nine of those seem to have come from Rose, including presumptively the two described as having two pronuclei. Rose was invited to be a co-author, but declined for reasons unknown. The source of the remaining nine eggs is unknown, but may have come from Oldham General Hospital. The Acknowledgements thank ‘for their help’ Drs. C Abberley, G Garrett and L Davies, all of Oldham General. John Webster, a later gynaecological colleague of Steptoe, having consulted his colleague John Battie, writes of these:

“Cyril Adderley, not Abberley, was the Group Pathologist in Oldham. Geoff Garrett ... was also a pathologist there ... there was no L. Davies there but a John Davies, a haematologist, and a Vincent John Davies, a histologist ... and my money would go on V. J. as I think a histologist rather than a haematologist would have been of more help to him ...?”

The first two of these have elsewhere been described as helpful in setting up the embryology laboratory in Oldham, largely through the provision or loan of equipment required locally for egg maturation and fertilization, so their involvement in direct provision of eggs seems unlikely.

The Nature paper also supports Oldham as the source of the remaining eggs: ‘Some eggs were transported from Oldham to Cambridge’ (Edwards et al., 1969a), and in his retrospective 1980 account of the events, Edwards says that at Oldham they began to repeat the experiment:

“Twelve women whose ovaries had to be removed [presumably laparoscopically] for serious medical conditions provided us with the necessary eggs over the next few months. We fertilized many more eggs and were able to make detailed examinations of the successive stages of fertilization (Edwards and Steptoe, 1980, pp. 82–83).”

So it seems reasonable to conclude that those eggs described in the paper as ‘undergoing fertilization’ were provided in roughly equal numbers by Rose and Steptoe.

However, with Steptoe on board, Rose no longer featured as a supplier of eggs (Edwards and Steptoe, 1980, p. 81). Whilst the initial attraction of laparoscopy for Edwards had been the recovery of capacitated spermatozoa from the oviduct, once working with Steptoe he rapidly saw the wider possibilities for recovery of in-vivo matured eggs from the ovary (Steptoe, 1968). Indeed, the 1969 paper includes the following statement:

“Problems of embryonic development are likely to accompany the use of human oocytes matured and fertilized *in vitro*. When oocytes of the rabbit and other species were matured *in vitro* and fertilized *in vivo*, the pronuclear stages appeared normal but many of the resulting embryos had sub-nuclei in their blastomeres, and almost all of them died during the early cleavage stages ... When maturation of rabbit oocytes was started *in vivo* by injecting gonadotrophins into the mother, and completed in the oviduct or *in vitro*, full term rabbit fetuses were obtained (Edwards et al., 1969a, pp. 634–635).”

The paper goes onto discuss how use of hormonal priming to stimulate intra-follicular egg maturation might be achieved and reports: ‘Preliminary work using laparoscopy has shown that oocytes can be recovered from ovaries by puncturing ripening follicles *in vivo* ...’.

Through these preliminary collaborative studies, Edwards and Steptoe were already building a research partnership. Although both were very different personalities, and brought very different skills to the project, they shared energy, commitment and vision. Each was also marginalized by his professional peers, a marginalization that also perhaps helped to cement their partnership (Johnson et al., 2010).

With the paper’s publication, announced to the media on St Valentine’s day (Anon, 1969), all hell was let loose. The impossible tangle of TV cables and pushy reporters trying to force their way up the stairs to the fourth floor laboratories proved a major disruption to the Physiological laboratory in general and to the members of the Marshall laboratory in particular. It was something that was to recur episodically over the next 10 years.

### The battles begin

But 1969 seemed to be a good year for Edwards. Not only did IVF succeed at long last, and his partnership with Steptoe seemed set to flourish, but also so impressed were the Ford Foundation with his work that in late 1968 they had established, at Austin’s prompting (Holmes, 1968), an endowment fund with the University of Cambridge to cover the salary cost of a Ford Foundation Readership (a half-way step to a professorship; Hankinson, 1968). Elated by his promotion and their achievement, Edwards and Steptoe pressed on, the latter’s laparoscopic skills coming to the fore, first in 1970 with the collection of in-vivo matured eggs from follicles after mild hormonal stimulation (Steptoe and Edwards, 1970), and then achieving regular fertilization of these eggs and their early development through cleavage to the blastocyst stage (Edwards et al., 1970; Steptoe et al., 1971). So well was the work going that in late 1970 and early 1971 they confidently applied to the UK Medical Research Council for funding.

However, any illusions that Edwards may have had that their achievements would prove a turning point in his fortunes were soon shattered. The hostility of much of the media coverage to his work in 1969 heralded the dominant pattern of scientific and medical responses for the next 10–15 years and resulted just 2 months later in the MRC rejecting the grant application (Johnson et al., 2010). The practical consequences of this rejection were profound – both psychologically and physically – not least that for the next 7 years, Edwards and Purdy shuttled on the 12-h-round trip between Cambridge and Oldham, Greater Manchester, paradoxically just north of his schoolboy haunts of Gorton, where Steptoe and he set up a small laboratory and clinic in Dr. Kershaw’s cottage hospital, all the while leaving Ruth and his five daughters in Cambridge.

The professional attacks on Edwards and his work took a number of forms (Johnson et al., 2010), and one must try to make a mental time trip back to the 1960s and 1970s to understand their basis. Despite the nature of the political and religious battles to come, his scientific and medical colleagues did not focus on the special status of the human embryo as an ethical issue. Ethical issues were raised professionally, but took quite a different form. It is perhaps difficult now to comprehend the complete absence of infertility from the consciousness of most gynaecologists in the UK at the time, of whom Steptoe was a remarkable exception (Edwards, 1996, p. 436; Edwards and Steptoe, 1980, pp. 11–15). Indeed, even Edwards’ strong commitment to treating infertility came to the fore only after he had teamed up with Steptoe, his previous priority being the study and prevention of genetic and chromosomal disorders. In the several reports from the Royal College of Obstetricians and Gynaecologists and the MRC during the 1960s examining the areas of gynaecological ignorance that needed academic attention, infertility simply did not feature (MRC, 1969; RCOG, 1967). Overpopulation and family planning were seen as dominant concerns and the infertile were ignored as, at best, a tiny and irrelevant minority and at worst as a positive contribution to population control. This was a values system that Edwards did not accept (Edwards and Sharpe, 1971, p.87), and the many encouraging letters he received from infertile couples spurred him on and provided a major stimulus to his continued work later, despite so much professional and press antagonism. For his professional colleagues, however, the fact that infertility was not seen as a significant clinical issue meant that any research designed to alleviate it was viewed not as experimental treatment, but as using humans in experiments. Given the sensitivity to the relatively recent Nazi ‘medical experiments’, the formal acceptance of the Helsinki Declaration (1964; Hazelgrove, 2002) and the public reaction and disquiet surrounding the recent publication of Human Guinea-pigs (Pappworth, 1967), this distinction was critical. The MRC, in rejecting the grant application, took the position that what was being proposed was human experimentation, and so were very cautious, emphasizing risks rather than benefits, of which they saw few if any (Johnson et al., 2010).

Edwards and Steptoe were also attacked for their willingness to talk with the media. It is difficult nowadays, when the public communication of science is embedded institutionally, to understand how damaging to them this was. The massive press interest of the late 1960s was unabated in the ensuing years, and so Edwards was faced with a choice: either he could keep his head down and allow press fantasies and speculations to go unanswered and unchallenged, or he could engage, educate and debate. For him this was no choice, regardless of the consequences professionally (Gardner and Johnson, 2011). His egalitarian spirit demanded that he trust common people’s common sense. His radical political views demanded that he fought the corner of the infertile: the underdog with no voice. The Yorkshireman in him relished engagement in the debate and argument. In Edwards and Sharpe (1971), he sets out his reasons for public engagement and acknowledges the risk to his own interests:

“Scientists may have to make disclosures of their work and its consequences that run against their immediate interests; they may have to stir up public opinion, even lobby for laws before legislatures.”

And risk it was. One of the scientific referees on their MRC grant application started his referee’s report declaring the media exposure distasteful:

“Dr. Edwards feels the need to publicise his work on radio and television, and in the press, so that he can change public attitudes. I do not feel that an ill-informed general public is capable of evaluating the work and seeing it in its proper perspective. This publicity has antagonised a large number of Dr. Edwards’ scientific colleagues, of whom I am one (Johnson et al., 2010).”

Edwards’ pioneering role in the public communication of science proved to be disadvantageous to his work.

The Edwards and Sharpe (1971) paper is a tour de force in its survey of the scientific benefits and risks of the science of IVF, in the legal and ethical issues raised by IVF, and in the pros and cons of the various regulatory responses to them. It sets out the issues succinctly and anticipates social responses that were some 13–19 years into the future. Edwards built on his strong commitment to social justice based on a social ethic in subsequent years, as he engaged at every opportunity with ethicists, lawyers and theologians, arguing, playing ‘devil’s advocate’ (literally, in the eyes of some), and engaging in what would now be called practical ethics as he hammered out his position and felt able to fully justify his instincts intellectually.

**Figure 13:**
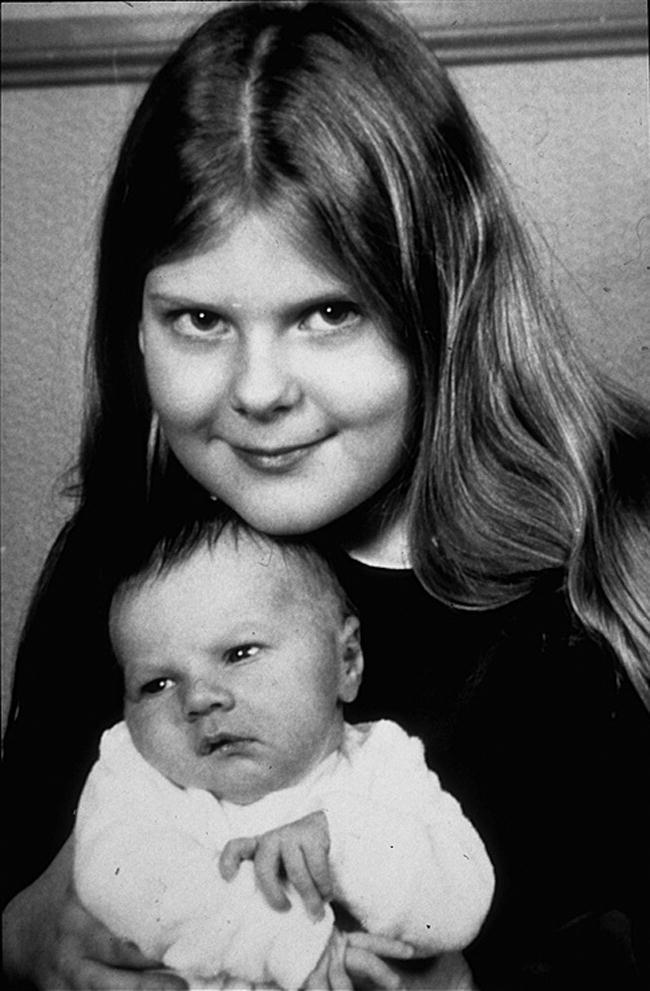
Louise Brown holding the 1000th Bourn Hall baby 1987 (courtesy Bourn Hall Clinic).

However, the establishment was, with few exceptions, unwilling to engage seriously in ethical debates (Edwards, 1974; Jones and Bodmer, 1974) in advance of the final validation of IVF that was to come in 1978 with the birth of Louise Brown (Figure 13; Edwards and Steptoe, 1978). Only then did the UK social, scientific and medical hierarchies, such as the MRC, the British Medical Association, the Royal Society and Government move gradually from their almost visceral reactions against IVF and its possibilities to serious engagement with the issues (Johnson and Theodosiou, 2011). Then, to their credit, both the MRC and the Thatcher Government of the time came on board, but it was not until 1989, 24 years after Bob’s 1965 visionary paper in the Lancet, that the UK Parliament finally gave its stamp of approval to his vision, and then only after a fierce battle lasting some 11 years (Johnson and Theodosiou, 2011; Mulkay, 1997).

## Discussion

This paper describes some of the early years of Edwards’ life and work, in order to provide a context for the events leading up to the 1969 Nature paper describing IVF and the final validation of the claims made in that paper with the birth of Louise Brown in 1978. It is evident even from the earliest stages of his late entry into research that Edwards is a man of extraordinary energy and drive, qualities sustained throughout his long career, witnessing his prodigious output of papers between 1954 and 2008 (Gardner and Johnson, 2011). Indeed, several of the referees on the unsuccessful MRC grant application specifically criticized his ‘over-enthusiasm’, doubting that he could achieve the programme he sets out therein as ‘too ambitious’ (Johnson et al., 2010). Tenacity of purpose comes through clearly in Edwards’ work, a trait he is inclined to attribute to his Yorkshire origins, but which may also be fuelled by his working-class determination to show himself as good as the next (wo)man.

The influence of Waddington’s Edinburgh Institute, of Waddington himself, and of his superviser, Alan Beatty, on Edwards’ interests and values is also clear from the dominant role that developmental genetics played in his thinking, especially until the time he met Steptoe. Indeed, from examination of Edwards’ papers and interests, his passionate conversion to the cause of the infertile seems directly attributable to Steptoe’s influence. Admittedly, Edwards’ forays into immuno-reproduction did involve consideration of immunological causes of infertility, but these were more usually of interest to him as models for developing new contraceptive agents. Indeed, Edwards was as captured as most reproductive biologists of the time by the 1960s’ consensus on the need for better methods of world population control. This position was understandable given the reality of those concerns, as is demonstrated now in the problem of global warming that is attributable at least in part to a failure to control population growth. It is a measure of his imagination and empathy that he could grasp so rapidly Steptoe’s understanding of the plight of the infertile and so flexibly incorporate this understanding into his plans. That empathy clearly reflects his under-privileged origins, his espousal of the cause of the junior, the disadvantaged, the ill-informed and the underdog being a thread running through his career. Edwards can be very critical, but I have found no one who can remember him ever being nasty or vindictive. Even when he disagrees with some one passionately, he never loses his respect for them as people. That Steptoe tapped into this sentiment is clear.

The way in which Edwards met Steptoe has been absorbed into folklore, but an examination of the evidence seems to warrant some revision to commonly held later reminiscences. It remains uncertain exactly which publication(s) by Steptoe it was that Edwards read in 1967, but seems likely that he did read Steptoe’s book. Thus, it was spermatozoa, not eggs, that were exercising Edwards in 1967, and it was the problem of sperm capacitation, not egg retrieval, to which Steptoe and his laparoscope seemed to offer a solution. The book is the only place that this issue is specifically addressed. Their actual meeting at the Royal Society of Medicine is also re-evaluated: Edwards was an invited speaker lecturing about his work on immuno-reproduction, so, paradoxically, what has been seen as a side track to his main work, was, albeit serendipitously, the reason for their actual meeting.

**Figure 14:**
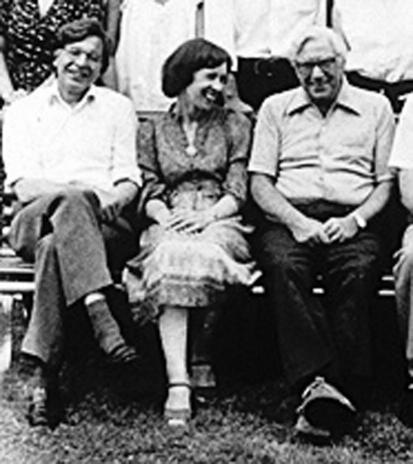
Edwards, Purdy and Steptoe at Bourn Hall 1981 (courtesy Bourn Hall Clinic).

The early collaboration between them involved the recovery of ovarian biopsies, just like those Rose and others had been providing. However, the attractions of preovulatory follicular egg recovery were already clear to them both by the end of 1968, and became, with embryo replacement, the central planks of their partnership. Steptoe and Edwards were in many ways an unlikely partnership. Their personal styles were very different, and there are clear hints in his writings that Edwards found their early days together difficult. But like most successful partnerships, their differences were sunk in a mutual respect for the other’s pioneering skills and willingness to take on the established conventions. In Jean Purdy, they also had a partner who smoothed the bumps on the path of their work together (Figure 14).

However, it remains Edwards’ extraordinary foresight that marks him out so distinctively. His combination of vision and intellectual rigor is evident not just in his work on stem cells, PGD and, with Steptoe, infertility, but also in his pioneering work in the public communication of science, in how ethical discourse about reproduction is conducted, and in consideration of regulatory issues. The epithet ‘the father of Assisted Reproductive Technology’ is surely deservedly appropriate.

## Figures and Tables

**Table 1:** Key points in the programme of research laid out in the Discussion to Edwards’ 1965 Lancet paper.

1	Studies on non-disjunction of meiotic chromosomes as a cause of aneuploidy in humans^a^
2	Studies on the effect of maternal age on non-disjunction in relation to the origins of trisomy 21^a^
3	Use of human eggs in IVF to study fertilization
4	Study of culture methods for human eggs fertilized *in vitro*
5	Use of priming hormones to increase the number of eggs per woman available for study/use
6	Study of early IVF embryos for evidence of (ab)normality – especially aneuploidies arising prior to or at fertilization^a^
7	Control of some of the genetic diseases in man^a^
8	Control of sex-linked disorders by sex detection at blastocyst stage and transfer of only female embryos^a^
9	Intra-cervical transfer of IVF embryos into the uterus
10	Use of IVF embryos to circumvent blocked tubes^b^
11	Avoidance of a multiple pregnancy (as observed after hormonal priming and in vivo insemination) by transfer of a single IVF embryo

a Five aims relating specifically to genetic disease.

b One aim relating specifically to infertility relief.

**Table 2:** Edited record from the RSM Endocrinology Section: General Minutes, 1946–1975 (ref: RSM/J/19/4/1) p.365 (with permission).

A joint meeting of the SECTION OF ENDOCRINOLOGY of the Royal Society of Medicine with the SOCIETY FOR THE STUDY OF FERTILITY was held at 1 Wimpole Street, W.1., Wednesday, 28 February 1968, at 10.00 am.
The meeting was attended by approximately 127 Fellows, members and guests and the programme was as follows:
‘FERTILITY AND INFERTILITY’
10.00: Chairman’s opening remarks
10.10: Sperm capacitation. C.R. Austin, Department of Embryology, Cambridge
10.35: Immunological aspects of infertility. R.G. Edwards, Department of Physiology, Cambridge
11.00: Coffee
11.30: The rating of semen quality by chemical methods. Dr. T. Mann, Department of Physiology of Reproduction, Cambridge
11.55: Endocrine studies in women with secondary amenorrhoea. Prof. Ivor H. Mills and R.J. Wilson, Department of Investigative Medicine, Cambridge
12.20: Some investigation in male hypogonadism. Prof. F.T.G. Prunty, Department of Chemical Pathology, St Thomas’s Hospital Medical School, London
12.45: Lunch
2.15: A gonadotrophin stimulation test for ovarian responsiveness. G.I.M. Sawyer, University College Hospital Medical School, London
2.40: Factors affecting the response to clomiphene therapy. D. Ferriman, A.W. Purdie and M. Corns, North Middlesex Hospital, London
3.05: Comparison of clomiphene and F.S.H. for treatment of anovulation. A.D. Tsapoulis and A.C. Crooke, Department of Clinical Endocrinology, Birmingham
3.30: TEA
4.00: Time cause of urinary oestrogen exoetion after various schemes of Pergonal therapy. J.K. Butler, G.D. Searle and Co., High Wycombe, Bucks
4.25: Recent developments in the control of fertility. Sir Alan S. Parkes, Cambridge
4.50: General discussion
[signed] C.L. Cope 26/6/1968 [pasted in] 26th June 1968

**Table 3:** Steptoe’s papers from 1967 and earlier.

Publication	Title	Type of publication	Location in Cambridge; date of arrival
Steptoe (1967a)	Laparoscopy in Gynaecology	Book	University library; March 1967
Steptoe (1967b)	A new method of tubal sterilisation	Conference proceedings (Stockholm)	Physiology library; Arrival date unknown, published in November 1967
Steptoe (1967c)	Laparoscopic studies of ovulation, its suppression and induction, and of ovarian dysfunction	Conference proceedings (Sydney)	University library; May 1968
Steptoe (1966)	The fifth freedom	BMJ letter (22 January), 234	Physiology library; January 1966
Steptoe (1965)	Gynaecological endoscopy – laparoscopy and culdoscopy	Paper in J Obstet Gynaecol Br Commonw 72, 535–543	University library; August 1965 (moved to Clinical School library after 1973)

**Table 4:** Summary of data from Edwards et al. (1969a).

Egg characteristic	Experimental group	Control group
Assigned	56	17
Surviving	54/56	17/17
Matured to metaphase II	34/54	7/17
Some evidence of sperm penetration	18/34	–
Spermatozoon within the zona pellucida	6/18	–
Spermatozoon inside zona pellucida (c.7 h post-insemination)	5/18	–
Evidence of pronuclei (c.11 h post-insemination)	7/18	0/7
No. with two pronuclei	2/18	–
